# Supplementary figures and images for: Prediction of PM2.5 concentration based on a CNN-LSTM neural network algorithm
Source: PeerJ. 2024 Aug 6;12:e17811. doi: 10.7717/peerj.17811 (PMC11313410; doi:10.7717/peerj.17811)

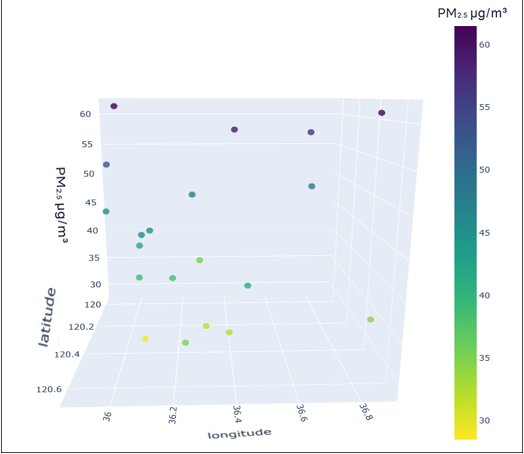

Supplement: Supplemental Information 6 [file peerj-12-17811-s006.png]

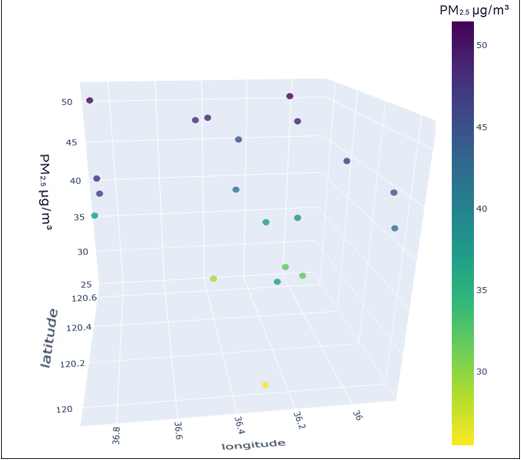

Supplement: Supplemental Information 7 [file peerj-12-17811-s007.png]

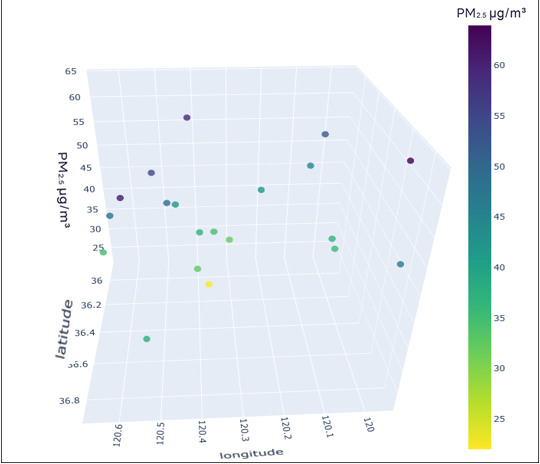

Supplement: Supplemental Information 8 [file peerj-12-17811-s008.png]

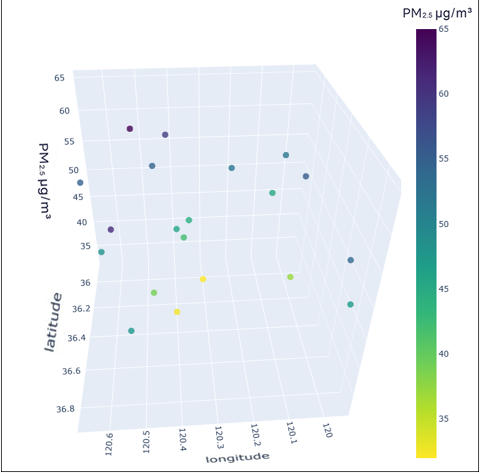

Supplement: Supplemental Information 9 [file peerj-12-17811-s009.png]

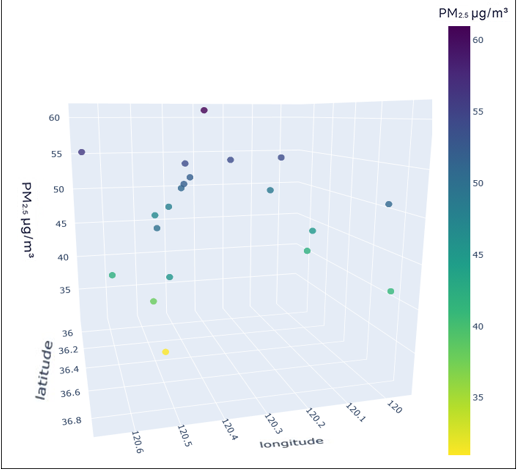

Supplement: Supplemental Information 10 [file peerj-12-17811-s010.png]

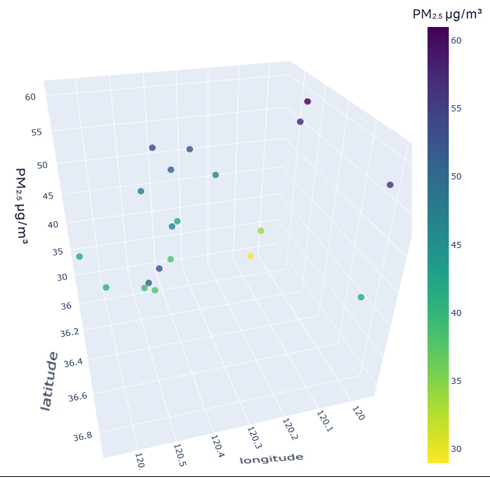

Supplement: Supplemental Information 11 [file peerj-12-17811-s011.png]

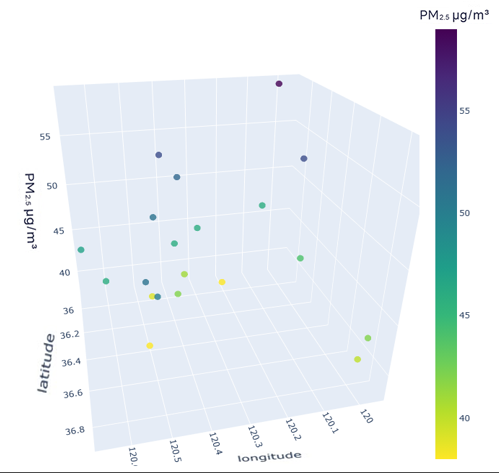

Supplement: Supplemental Information 12 [file peerj-12-17811-s012.png]

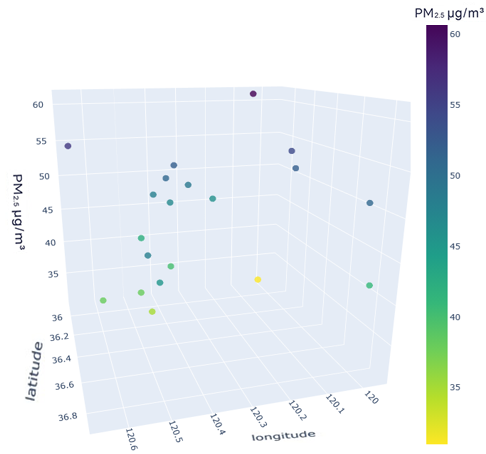

Supplement: Supplemental Information 13 [file peerj-12-17811-s013.png]

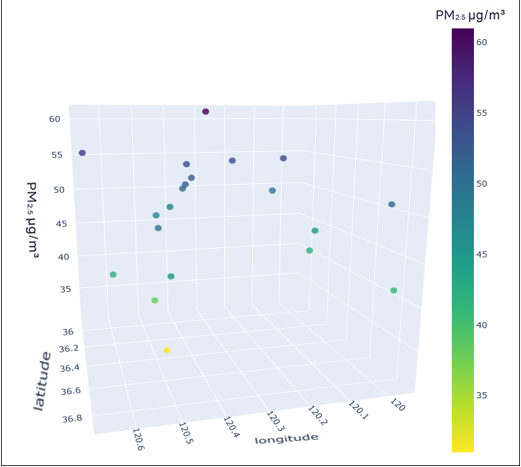

Supplement: Supplemental Information 14 [file peerj-12-17811-s014.png]

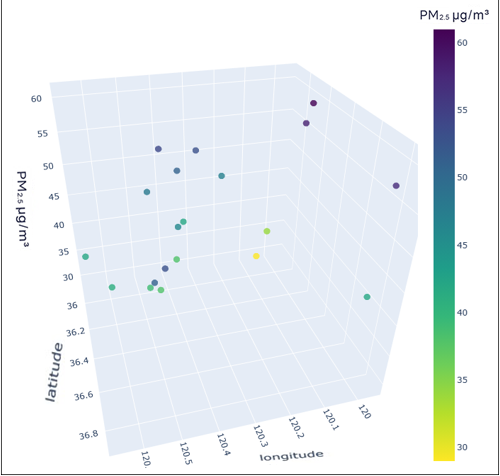

Supplement: Supplemental Information 15 [file peerj-12-17811-s015.png]

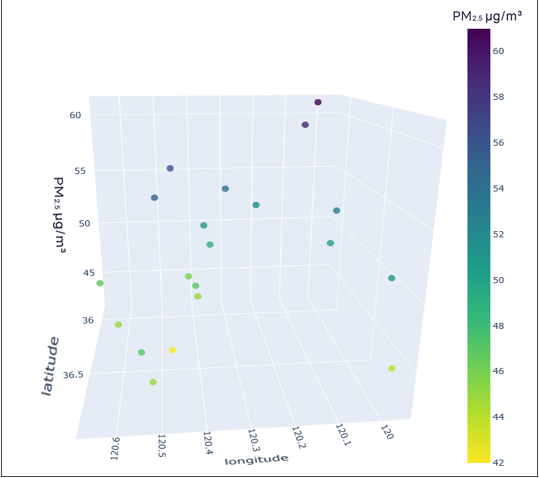

Supplement: Supplemental Information 16 [file peerj-12-17811-s016.png]
